# Acupressure and Cognitive Training Can Improve Cognitive Functions of Older Adults With Mild Cognitive Impairment: A Randomized Controlled Trial

**DOI:** 10.3389/fpsyg.2021.726083

**Published:** 2021-11-17

**Authors:** Jingxian Sun, Hui Zeng, Lu Pan, Xiaosong Wang, Mengjiao Liu

**Affiliations:** ^1^School of Nursing, Nanjing University of Chinese Medicine, Nanjing, China; ^2^Xiangya School of Nursing, Central South University, Changsha, China; ^3^Second Xiangya Hospital, Central South University, Changsha, China; ^4^Jiangxi Provincial Cancer Hospital, Nanchang, China

**Keywords:** older adult, mild cognitive impairment, acupressure, cognitive training, cognitive function

## Abstract

**Background:** Given the limited effectiveness of pharmacological treatments in mitigating cognitive decline in individuals with mild cognitive impairment (MCI), there is a pressing need for developing effective non-pharmacological intervention programs to counteract MCI-related cognitive decline. Acupressure and cognitive training are safe and cost-effective; however, evidence of the effect of acupressure or the combined effect of acupressure and cognitive training on cognitive functions of older adults with MCI is limited.

**Objective:** To evaluate both the individual and combined effects of acupressure and cognitive training on cognitive functions of older adults with MCI.

**Methods:** One hundred and eighty older adults with MCI were recruited and randomly assigned to combined acupressure and cognitive training group (*n* = 45), acupressure group (*n* = 45), cognitive training group (*n* = 45), or control group (*n* = 45). Participants in the experimental groups received self-administered and group-based training sessions, while those in the control group received routine community education. The intervention lasted for 6 months. The cognitive functions of all the participants were assessed at multiple stages, including pre-intervention, at the end of the third and sixth months.

**Results:** One hundred and fifty-one participants completed the study, and all participants analyzed in intervention groups completed at least 85% of all practice sessions recommended. Repeated measures analysis of variance of the scores of Mini-Mental State Examination (MMSE) and Montreal Cognitive Assessment (MoCA) at different time points among the four groups revealed that the group effect, time effect, and interaction effect were all significant (*p* < 0.01). Pairwise comparisons with Bonferroni correction showed that the scores of MMSE and MoCA in acupressure group, cognitive training group, and combined group were significantly raised compared with control group (*p* < 0.01). Compared with acupressure or cognitive training groups, the scores of MMSE and MoCA in combined group were significantly higher (*p* < 0.05). The scores of MMSE and MoCA in acupressure group had no significant differences with those in cognitive training group (*p* > 0.05).

**Conclusion:** Acupressure and cognitive training both could improve the cognitive functions of older adults with MCI, and when used together, the effects were enhanced.

**Clinical Trial Registration:** This study was registered in the Chinese Clinical Trial Registry (No.ChiCTR2100049955).

## Introduction

Mild cognitive impairment (MCI) is a condition in which individuals demonstrate cognitive impairment with minimal impairment of instrumental activities of daily living (IADL) (Petersen et al., 2018). MCI is an intermediate state between normal cognition and dementia. Persons with MCI are at higher risk of progressing to dementia than age-matched controls, and the cumulative dementia incidence was 14.9% in individuals with MCI older than age 65 years followed for 2 years (Tangalos and Petersen, 2018). According to the 2018 American Academy of Neurology (AAN) guideline on MCI, MCI prevalence was 6.7% for ages 60–64, 8.4% for 65–69, 10.1% for 70–74, 14.8% for 75–79, and 25.2% for 80–84. Thus, there is significant public health value in identifying MCI early and intervening as soon as possible to prevent or postpone the transition from MCI to dementia.

To date, pharmacological interventions have been suboptimal in slowing the cognitive decline and preventing the transition of patients with MCI to dementia (Andrieu et al., 2015; Petersen et al., 2018). Therefore, there are growing interests in the effectiveness of non-pharmacological interventions. Cognitive training is an increasingly popular, non-pharmacological intervention for improving cognitive functioning in neurodegenerative diseases and healthy aging (Van Balkom et al., 2020). Cognitive training involves cognitive exercises that target specific cognitive abilities and neural networks to potentially improve cognitive functioning through neuroplasticity (D’Antonio et al., 2019). Systematic reviews show evidence that cognitive training can be effective in improving various aspects of objective cognitive functioning; memory performance, executive functioning, processing speed, attention, fluid intelligence, and subjective cognitive performance (Reijnders et al., 2013; Hill et al., 2017; Sherman et al., 2017). The 2018 American Academy of Neurology (AAN) guideline on MCI recommended cognitive training as a non-medication approach to improve cognitive function (Petersen et al., 2018).

Combining cognitive training with other interventions might greatly increase the likelihood of cognitive benefit. Recent studies have indicated that there appears to be merit in multicomponent composite approaches involving cognitive training and other interventions such as physical activity (Karssemeijer et al., 2017; Gavelin et al., 2021), aerobic exercise (Mirza and Yaqoob, 2018; Park H. et al., 2019), transcranial direct current stimulation (Lawrence et al., 2018) and risk factor modification (Xu et al., 2020) for improving cognitive performance in older adults with MCI, possibly offering more benefit than a single-bout intervention approach. A meta-analysis showed that multicomponent and multidomain forms of intervention might prompt recruitment of alternate neural processes as well as support primary networks to meet task demands simultaneously (Sherman et al., 2017).

Acupressure, a non-invasive technique, involves the use of fingers, thumbs, knuckles, or an appropriate acupressure tool to apply pressure to the acupoints (Schlaeger et al., 2017). A 1-year randomized controlled trial conducted by Zeng et al. (2016) showed that acupressure improved the older adults’ sleep quality as well as cognitive functions. McFadden et al. (2011) found that acupressure was associated with enhanced working memory function in participants suffering from cognitive impairment following traumatic brain injury. According to Traditional Chinese Medicine (TCM), acupressure treatment can stimulate the related meridians and reproduce an effect usually defined as “De Qi” to correct the imbalance of Qi in the body of patients with MCI (Smith et al., 2020). From the biomedical perspective, acupressure may stimulate the nervous system and positively modulate the regulation of serum cortisol, serotonin, endorphin, and the activity of different neurotransmitters (Moyer et al., 2011; Hmwe et al., 2015; Zullo et al., 2020). There are no reports of adverse reactions or sequelae from the use of any acupressure techniques, thus acupressure is a safe therapy. So far, the effect of acupressure on cognitive functions of subjects with MCI has not been reported.

Acupressure and cognitive training are two promising non-pharmacological interventions. Acupressure may stimulate the nervous system and alter physiological functions since the majority of acupressure points are either connected to, or located near neural structures (Zullo et al., 2017), while cognitive training improves selective brain function intensively (De Marco et al., 2018; Park J. et al., 2019). Hence, combined acupressure and cognitive training may yield a synergistic effect on cognition by complementary strengthening of different neural functions. However, the efficacy of the combined intervention compared to a single acupressure or a single cognitive training intervention remains unclear in older adults with MCI. Given the uneven distribution of medical resources worldwide and the vast elderly population, feasible, safe, and cost-effective interventions are required to intervene MCI. Therefore, this study aimed to evaluate the effects of acupressure and cognitive training, both individual and combined, on cognitive functions of older adults with MCI. Two hypotheses derived from the aim of this study: (i) acupressure, as well as cognitive training, would have beneficial effect for cognitive functions of older adults with MCI, and (ii) the combined effect of acupressure and cognitive training would outperform acupressure or cognitive training alone in improving cognitive performance of older adults with MCI.

## Methods

### Study Participants

When the mean values of four groups were compared, the effect sizes from small to large were 0.01, 0.05, 0.10, 0.20, and 0.30 successively (Denise and Bernadette, 1999). Based on the effect size of a previous study (Zeng et al., 2016), we assumed a moderate effect size of this study (*f* = 0.07), a minimum of 37 samples were required for each group, as these numbers could reach 80% power at a 5% significance level (Denise and Bernadette, 1999). To allow for a drop-out rate of 20%, a total of 180 participants should be recruited for this RCT (45 participants in each group with 1:1:1:1 allocation).

The study was conducted at a community in a city of Central China, from April 2019-January 2020.

The recruit announcements were posted on the community health service center, as well as billboards in the community. Older adults who were over 60 years old and had cognitive complaints could come for an evaluation in the community health service center. We recruited 1,653 community-dwelling older adults from April 2019–June 2019. All participants were evaluated through a clinical interview conducted by a specialist in cognitive disorders. The following criteria, established by Petersen (2016), was used for clinical diagnosis of amnestic MCI: (1) cognitive complaint by the patient, the patient’s informant, or the physician, (2) cognitive decline which is not normal for age, (3) essentially normal functional activities, (4) no dementia, and (5) memory impaired. One hundred and ninety-eight older adults were diagnosed with amnestic MCI. Eighteen were excluded according to the inclusion criteria and exclusion criteria. The inclusion criteria for the study included elderly who (i) were over 60 years old, (ii) were diagnosed with amnestic MCI, and (iii) consented to participate in the research. The exclusion criteria included elderly who (i) had severe visual or hearing impairment; (ii) had pre-existing musculoskeletal conditions, which prohibited the application of acupressure at the selected points; or (iii) had suffered from a neurological disorder (e.g., severe head trauma), psychiatric disease (e.g., major depression), or any other major medical disease that could potentially compromise their cognition.

After obtaining written consent from the 180 eligible participants, they were randomly assigned to combined acupressure and cognitive training group, acupressure group, cognitive training group, or control group, with 45 participants in each group.

### Interventions

#### Acupressure

**FIGURE 1:**
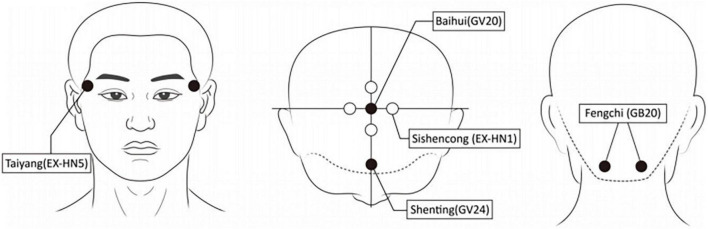
Acupoints.

The acupressure points were chosen according to the effective acupuncture points for the treatment of MCI. Acupuncture involves the insertion of fine needles into different parts of the body to correct the imbalance of energy in the body (Smith et al., 2020). Acupressure is based on the same paradigm as acupuncture. When acupressure is applied, the same points are activated by using hands and fingers as applied by acupuncture. So far, there is no unified and standard acupuncture point for acupuncture treatment of MCI (Hou et al., 2020). The combinations of points were usually used to achieve greater effects for the treatment of MCI. Systematic reviews and meta-analyses (Deng and Wang, 2016; Kim et al., 2019; Li et al., 2020) indicated that the most frequently used acupuncture points for the treatment of MCI were Baihui (GV20), Fengchi (GB20), Shenting (GV24), and Sishencong (EX-HN1). Taiyang (EX-HN5), which can bring relaxation and refreshment as an acupressure point, is an ideal acupoint when treating disorders affecting the head (Lu et al., 2021). Therefore, the following special acupoints were chosen in the present study: Baihui (GV20), Fengchi (GB20), Shenting (GV24), Sishencong (EX-HN1) and Taiyang (EX-HN5), as shown in Figure 1.

Baihui (GV20) is located at the middle of the vertex, on the line connecting the apexes of the two ears; Fengchi (GB20) is located at the posterior lateral area of the neck, in the fossa between the superior margins of the trapezius and sternocleidomastoid muscles; Shenting (GV24) is located on the head, 0.5 cun (one cun is the width of the participant’s own thumb according to TCM) directly above the midpoint of the anterior hairline; Sishencong (EX-HN1) includes a group of four points which are located on the vertex of head, each 1 cun away from Baihui (GV20) in the four directions (anterior, bilateral, and posterior); and Taiyang (EX-HN5) is located at the point of intersection of the continuations of the eyebrow and the lower eyelid in the lateral direction, on the lateral border of the orbit.

For each acupoint, the middle finger pulp was gently placed on it, and the pressure was gradually increased. The pressure was strong and deep, depending on the tolerance of individual participants. The pressure was maintained for 10 s, followed by a clockwise movement for 30 s and anticlockwise for the next 30 s. Then, the pressure was released, and the point was massaged continuously for 15 s to relax. This process was repeated for each acupoint. According to our previous study (Zeng et al., 2016) and the tolerance of older adults, the total acupressure time was limited to no more than 20 min for one session, three sessions a day, and 5 days a week.

The acupressure protocol was explained to the participants in acupressure group and combined group in detail. Next, the participants attended intensive training once a week for the first 2 weeks. During the first training, researchers demonstrated the location of the acupoints, as well as the acupressure techniques, and conducted a group practice session. In the second round of training, participants were individually guided to make sure that each participant had mastered the correct acupoint locations and acupressure techniques. Then, the participants in acupressure group and combined group were subdivided into five subgroups, respectively, and participants who demonstrated good skills and organizational abilities were selected as team leaders to conduct subgroup practice five times a week (morning) from the third week. Additionally, the participants were also asked to practice acupressure at home by themselves twice a day, 5 days a week. Also, tabular diaries with tick or cross were requested to record daily practice by each participant. Systematic reviews (Deng and Wang, 2016; Kim et al., 2019) of acupuncture treatment for MCI indicated that the course of treatment time ranged from 30 days to 3 months. Thus, the intervention duration of this study was set to 6 months to observe the long-term effects of interventions.

#### Cognitive Training

At present, there is no unified content of cognitive training for the treatment of MCI. Based on a previous study (Liu et al., 2016), combing with our pre-investigation and the acceptance of the elderly in the community, the content of cognitive training in this study involved five aspects, including attention, memory, calculation, language, and executive functions. Table 1 describes the components and examples of the training. Materials on cognitive training were provided to participants in cognitive training group and combined group. The participants also attended an intensive training session once a week for the first 2 weeks to master the contents and methods of cognitive training. Then, the participants in cognitive training group and combined group were subdivided, respectively, into five subgroups; and subgroup leaders were selected to organize subgroup practice for approximately 1 h every day, 5 days a week from the third week.

**TABLE 1:** Components of cognitive training.

**Components**	**Training activities**	**Examples**	**Min/d**
Attention training	Schulte grid training Number repeat	Numbers of 1∼25 were filled at random in a table with 25 squares. The participants were asked to read the numbers in order of 1–25 with their fingers pointing at the numbers as quickly as possible. A random sequence of discrete numbers were read out, and the participants were asked to repeat the numbers immediately.	10
Memory training	Name recall Story retelling	Six famous faces were put on a sheet of paper, with names next to each face. The participants were asked to remember the names of the faces in 12 s (2 s for each face). When time is up, the names would be covered, and the participants were asked to recall the names according to the faces. The participants were asked to read a brief story prepared for them and retell the story again and again until they can retell it fluently. They were also encouraged to recall past life experiences, from their birth background to going to school, starting a family, having children and other memorable events they had experienced.	20
Calculation training	Logical ordering Doing arithmetic	The participants were asked to arrange objects in a particular logic order, for example, red, green, red, green. Simple calculation questions involving addition and subtraction were designed and divided into small units from easy to difficult, with each unit containing 10 calculation questions. The participants were asked to finish one small unit at a time.	10
Language training	Speaking and reading practice	Several meaningful pictures and beautiful articles were prepared for the participants. The participants were asked to choose a picture which they prefer and express their own thoughts. They were also encouraged to read the beautiful articles.	10
Executive functions training	Matchstick puzzles and a building block test	Matchsticks and building blocks were used as the main props. The participants were asked to follow the game instructions to arrange patterns and complete the following steps as required.	10

The intervention of all experimental groups lasted for 6 months, while the participants in control group received community health education, which included six sessions of health education lectures conducted by the community health staff. The lectures were conducted once a month, 1 h for each session, in the conference room of the community health service center. All the participants in control group attended the lectures as a whole. The session topics included how to maintain a healthy lifestyle, how to maintain good sleep habits, how to eat healthily, how to exercise effectively, how to cope with chronic diseases, and how to improve cognitive functions. The health education for control group also lasted for 6 months.

The following measures were taken to ensure proper compliance of participants in the experimental groups. First, a researcher or assistant oversaw several participants throughout the study. They conducted regular telephone interviews twice a week and home visits every 2 weeks to supervise the practice. The practice diaries of the participants were also checked during each home visit. Timely responses were provided to the questions or demands of the participants. Second, group leaders supervised the practice sessions of group members daily, and each group leader received a monthly allowance to enhance his/her sense of responsibility. Third, family members were encouraged to play a supervisory role.

#### Outcome Measures

The Mini-Mental State Examination (MMSE) and Montreal Cognitive Assessment (MoCA) were performed to test the cognitive abilities of the participants.

Zhang (1998) revised the Chinese version of the MMSE, which is a 30-point instrument with 11 items. The subjects’ cognitive functions were evaluated in five areas: orientation, memory, attention and calculation, language, and graphic simulation. Individuals could score from 0 to 30, with higher score indicating better cognitive performance. According to the original standardization’s studies conducted by Zhang (1998), the Cronbach’s alpha was 0.92, the test-retest reliability was 0.91 and the validity was 0.99.

The Chinese Beijing version of the MoCA scale (Yu et al., 2012) evaluates a range of cognitive functions, such as visuospatial/executive functions, attention, verbal memory registration and learning, 5-min delayed verbal memory, abstraction, and orientation with a total score of 0–30 (a higher score implies better cognitive function). The original standardization’s study showed that the Cronbach’s alpha was 0.818, and the test-retest reliability was 0.857 (Zhang and Liu, 2007). The total scores were consistent with the scores on the MMSE, and the correlation coefficient was 0.805 (Yu et al., 2012).

### Data Collection

The demographic data of the participants were collected at the baseline. The scores of MMSE and MoCA were measured before the intervention, at the end of the third and sixth months to evaluate the changes in the cognitive functions of the participants. Standardized instructions were adopted to assess cognitive functions individually in a quiet room free from distractions. All the data collectors were blind to the group allocation of the participants.

### Data Analysis

Statistical analysis was done using the Statistical Package for Social Sciences (SPSS) version 23.0 (SPSS Inc., Chicago, IL, United States). Measurement data were described through the means and standard deviations while counting data were described through frequency and percentage. Chi-square test, one-way analysis of variance (ANOVA) and pairwise comparisons with Bonferroni correction were done to examine the homogeneity of the baseline data on demographic features and cognitive functions among the groups. The repeated measures ANOVA was done to perform multiple comparison procedures by repeated measures. The multivariate test was used if the sphericity assumption was violated. Pairwise comparisons were done to detect the between-group differences, where the Bonferroni correction for multiple comparisons was applied. The α value was set at 0.05, and *p* values were two-tailed.

### Ethical Considerations

Ethics committee approval for this study was obtained from China Ethics Committee of Registering Clinical Trials and Central South University. Written informed consents were obtained from all the participants. Before signing the consent form, all potential study participants were provided with detailed information regarding the study, including its purpose, details regarding implementation, along with possible benefits and inconveniences. All participants were notified that they could leave the study at any stage without prejudice to their interests. All participants’ information was kept strictly confidential during the study period.

## Results

### Characteristics of Participants

**FIGURE 2:**
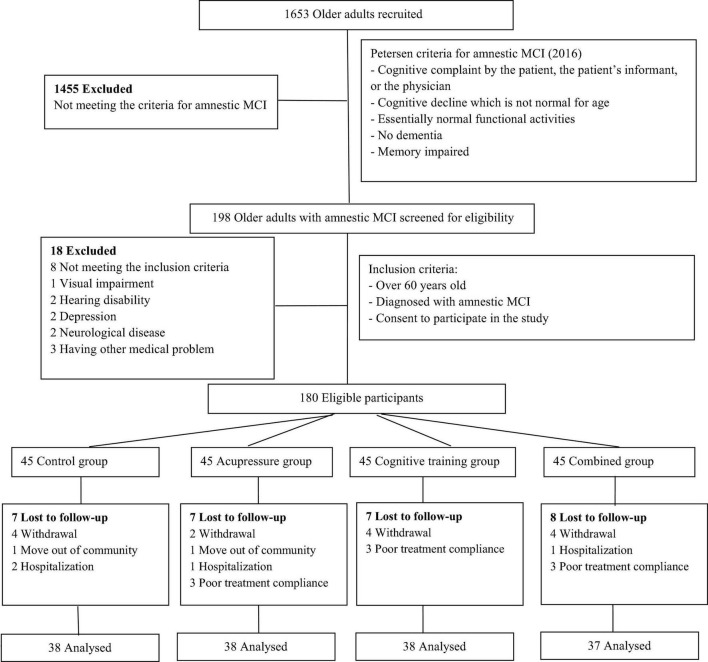
Flow chart showing participants in each group throughout the trial.

One hundred and fifty-one participants completed the study. Throughout the trial, the dropout rate was 16% (*n* = 29) because of withdrawal (*n* = 14), moving out of the community (*n* = 2), hospitalization (*n* = 4), and poor treatment compliance (*n* = 9). Figure 2 provides a flow chart showing participants in each group throughout the trial. According to the participants’ practice diaries, the group leaders’ reports, and the researchers’ monitoring records, all participants analyzed in intervention groups completed at least 85% of all practice sessions recommended (cognitive training group: 12 participants completed 85∼90%, 19 participants completed 90∼95%, seven participants completed 95∼100%; acupressure group: 10 participants completed 85∼90%, 18 participants completed 90∼95%, 10 participants completed 95∼100%; combined group: 16 participants completed 85∼90%, 14 participants completed 90∼95%, seven participants completed 95∼100%), and chi-square test showed that the differences among the intervention groups were insignificant (*p* > 0.05).

The baseline data for all one hundred and fifty-one participants indicated that the mean (SD) age was 70.83 (6.54) years, 35.76% were male, 64.24% were female, and 70.86% were married. Approximately three out of every four participants (76.16%) had chronic diseases, 64.24% had junior high school education or above, and 60.93% had an average monthly income of 2,000∼4,000 RMB ($308∼616). Chi-square test showed that there were no significant differences in demographical characteristics among the four groups (*p* > 0.05; Table 2). The baseline mean (SD) score of MMSE was 26.66 (1.78), and MoCA was 21.29 (2.23). One-way ANOVA and pairwise comparisons with Bonferroni correction both showed insignificant differences of the baseline scores of MMSE and MoCA among groups (*p* > 0.05).

**TABLE 2:** Basic demographical parameters and baseline characteristics of study population.

	**Acupressure group (*n* = 38)**	**Cognitive training group (*n* = 38)**	**Combined group (*n* = 37)**	**Control group (*n* = 38)**	***p*-value**
**Gender (%)**					
Male	10 (26.3)	18 (47.4)	16 (43.2)	10 (26.3)	0.108^a^
Female	28 (73.7)	20 (52.6)	21 (56.8)	28 (73.7)	
**Age (%)**					
60∼69	17 (44.7)	16 (42.1)	16 (43.2)	15 (39.5)	0.925^a^
70∼79	19 (50.0)	17 (44.7)	17 (46.0)	20 (52.6)	
≥80	2 (5.3)	5 (13.2)	4 (10.8)	3 (7.9)	
**Education (%)**					
Illiterate	0 (0.0)	1 (2.6)	1 (2.7)	2 (5.3)	0.874^a^
Elementary school	11 (29.0)	12 (31.6)	13 (35.1)	14 (36.8)	
Junior high school	12 (31.6)	13 (34.2)	13 (35.1)	11 (29.0)	
High school or above	15 (39.4)	12 (31.6)	10 (27.0)	11 (29.0)	
**Marital status (%)**					
Married	24 (63.2)	29 (76.3)	30 (81.1)	24 (63.2)	0.203^a^
Single/Divorced/Widowed	14 (36.8)	9 (23.7)	7 (18.9)	14 (36.8)	
**Income (RMB^c^/per month) (%)**					
None	9 (23.7)	5 (13.2)	9 (24.3)	7 (18.4)	0.653^a^
<2,000	0 (0.0)	1 (2.6)	2 (5.4)	2 (5.3)	
2,000∼4,000	20 (52.6)	26 (68.4)	22 (59.5)	24 (63.2)	
>4,000	9 (23.7)	6 (15.8)	4 (10.8)	5 (13.2)	
**Smoking (%)**					
Yes	7 (18.4)	6 (15.8)	1 (2.7)	4 (10.5)	0.106^a^
No	31 (81.6)	32 (84.2)	36 (97.3)	34 (89.5)	
**Drinking (%)**					
Yes	4 (10.5)	5 (13.2)	3 (8.1)	2 (5.3)	0.662^a^
No	34 (89.5)	33 (86.8)	34 (91.9)	36 (94.7)	
**Chronic diseases (%)**					
Yes	28 (73.7)	26 (68.4)	30 (81.1)	31 (81.6)	0.477^a^
No	10 (26.3)	12 (31.6)	7 (18.9)	7 (18.4)	
**Family history of stroke (%)**					
Yes	6 (15.8)	3 (7.9)	5 (13.5)	4 (10.5)	0.726^a^
No	32 (84.2)	35 (92.1)	32 (86.5)	34 (89.5)	
**Regular exercise (%)**					
Yes	28 (73.7)	32 (84.2)	25 (67.6)	28 (73.7)	0.413^a^
No	10 (26.3)	6 (15.8)	12 (32.4)	10 (26.3)	
MMSE*	26.87 (1.74)	26.71 (1.68)	26.68 (1.90)	26.37 (1.82)	0.669^b^
MoCA*	21.47 (2.22)	21.53 (2.00)	21.11 (2.22)	21.05 (2.50)	0.718^b^

*^a^p-values based on chi-square test.*

*^b^p-values based on one-way ANOVA.*

*^c^RMB is a monetary unit, and 1 United States dollar ≈ 6.47 RMB.*

*Data are expressed as mean (standard deviation). MMSE, Mini-Mental State Examination; MoCA, Montreal Cognitive Assessment.

### Results of the Outcome Measures

**FIGURE 3:**
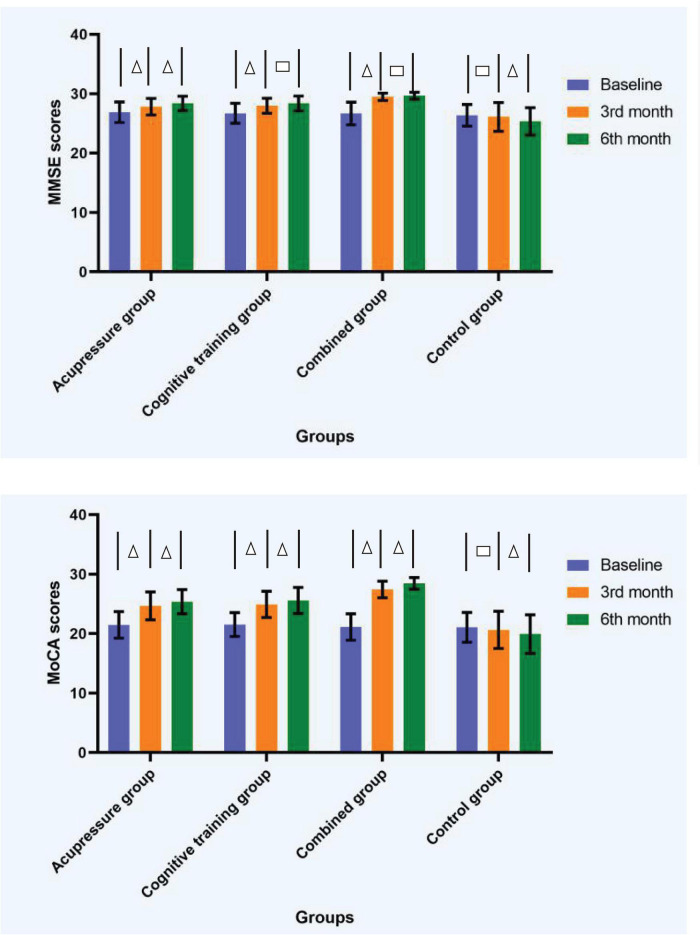
Diagrams showing measure outcomes at different time points among the four groups. △ The difference of scores of MMSE or MoCA between the two time points was significant (*p* < 0.05). □ The difference of scores of MMSE or MoCA between the two time points was insignificant (*p* > 0.05). Error bars based on standard deviation. MMSE, Mini-Mental State Examination; MoCA, Montreal Cognitive Assessment.

The mean scores of MMSE and MoCA of the four groups at each time point are shown in Table 3 and Figure 3. Repeated measures ANOVA of the scores of MMSE and MoCA at different time points among the four groups revealed that the group effect, time effect, and interaction effect were all significant (*p* < 0.01), as shown in Table 4.

**TABLE 3:** Mean scores of MMSE and MoCA of the four groups at each time point.

	**MMSE**			**MoCA**		
	**Baseline**	**3rd month**	**6th month**	**Baseline**	**3rd month**	**6th month**
Acupressure group	26.87 (1.74)	27.82 (1.39)	28.39 (1.20)	21.47 (2.22)	24.66 (2.35)	25.37 (2.03)
Cognitive training group	26.71 (1.68)	27.97 (1.26)	28.37 (1.24)	21.53 (2.00)	24.92 (2.20)	25.58 (2.20)
Combined group	26.68 (1.90)	29.49 (0.61)	29.68 (0.58)	21.11 (2.22)	27.43 (1.39)	28.46 (0.99)
Control group	26.37 (1.82)	26.11 (2.44)	25.34 (2.33)	21.05 (2.50)	20.63 (3.13)	19.92 (3.24)

*Data are expressed as mean (standard deviation). MMSE, Mini-Mental State Examination; MoCA, Montreal Cognitive Assessment.*

**TABLE 4:** Comparison of repeated measures among the four groups.

	**Between-group effect**			**Within-time effect**			**Interaction-effect**		
**Cognitive functions**	***F***	***p*-value**	**Effect size**	***F***	***p*-value**	**Effect size**	**F**	***p*-value**	**Effect size**
MMSE	23.734	<0.001	0.326	57.450	<0.001	0.440	20.354	<0.001	0.293
MoCA	37.166	<0.001	0.431	493.791	<0.001	0.871	36.5616	<0.001	0.427

*p-values based on repeated measures ANOVA. MMSE, Mini-Mental State Examination; MoCA, Montreal Cognitive Assessment.*

Pairwise comparisons with Bonferroni correction, which compared the average values of all repeated measurements among groups, showed that the scores of MMSE and MoCA in acupressure group, cognitive training group, and combined group were significantly raised compared with control group (*p* < 0.01). Compared with acupressure or cognitive training groups, the scores of MMSE and MoCA in combined group were significantly higher (*p* < 0.05). The scores of MMSE and MoCA in acupressure group had no significant differences with those in cognitive training group (*p* > 0.05). The Bonferroni-corrected *p*-values are listed in Table 5. Further comparisons of scores in MMSE and MoCA among groups within the same time point showed that, except for the insignificant difference between acupressure group and cognitive training group in scores of MMSE or MoCA within the same time point (*p* > 0.05), the other groups all differed from each other in scores of MMSE and MoCA within the time point of 3rd month or 6th month (*p* < 0.05).

**TABLE 5:** Pairwise comparisons of MMSE and MoCA scores among groups (*p*-value).

	**MMSE**				**MoCA**			
	**Acupressure group**	**Cognitive training group**	**Combined group**	**Control group**	**Acupressure group**	**Cognitive training group**	**Combined group**	**Control group**
Acupressure group	–	1.000	0.033	<0.001	–	1.000	0.002	<0.001
Cognitive training group	1.000	–	0.030	<0.001	1.000	–	0.007	<0.001
Combined group	0.033	0.030	–	<0.001	0.002	0.007	–	<0.001
Control group	<0.001	<0.001	<0.001	–	<0.001	<0.001	<0.001	–

*MMSE, Mini-Mental State Examination; MoCA, Montreal Cognitive Assessment. p-values based on Bonferroni correction.*

With respect to the interaction effect of time point and group, results indicated that the scores of MMSE significantly increased from the baseline to 3rd month time point for all the three intervention groups (*p* < 0.05). However, a further improvement in scores of MMSE from 3rd month time point to 6th month time point was observed only in the acupressure group (*p* < 0.05), while no significant differences emerged in the other training groups between the two time points (*p* > 0.05). The scores of MoCA significantly increased from the baseline to 3rd month time point, and from 3rd month time point to 6th month time point, for all the three intervention groups (*p* < 0.05). In regard to the control group, there was no significant difference in scores of MMSE or MoCA from the baseline to 3rd month time point (*p* > 0.05), however, significant decreases were observed from 3rd month time point to 6th month time point (*p* < 0.05).

## Discussion

Our findings are consistent with a growing body of research demonstrating the beneficial effects of cognitive training for patients with MCI (Feng et al., 2018; Li et al., 2019; Zhang et al., 2019b). Previous findings suggest that memory deficits in patients with MCI may emerge through a combined “loss” of medial temporal and frontoparietal functioning (Munro et al., 2015; Schnellbächer et al., 2020; Tang et al., 2021). Cognitive training is accompanied by significant and highly increases in activation in regions associated with the frontoparietal cognitive control network bilaterally (Hampstead et al., 2017; Raichlen et al., 2020; Simon et al., 2020). A systematic review (Miotto et al., 2018) indicated the association of cognitive improvement after cognitive training with both restorative (i.e., re-engaging the “typical” neural networks) and compensatory (i.e., engaging alternative neural networks that are not “typically” engaged) mechanisms.

This study also found that acupressure was comparably effective compared to cognitive training on improving cognitive functions of older adults with MCI. Acupressure is one of the major modalities of Traditional Chinese Medicine (TCM). TCM describes a state of health maintained by a balance of energy or Qi in the body (Xutian et al., 2009). According to TCM, Qi-Deficiency is a common feature in older adults with MCI (Deng et al., 2021). Acupressure treatment can stimulate the related meridians and reproduce an effect usually defined as “De Qi” to correct the imbalance of Qi in the body (Smith et al., 2020). From the perspective of modern medicine, it is suggested that acupressure may stimulate the nervous system since the fascial tissue of elderly people is susceptible to hands-on based conservative treatments (Zullo et al., 2020), and acupressure points are either connected to, or located near neural structures (Zullo et al., 2017). Manual stimulation of acupressure points has been shown to increase the production of serotonin and endorphin, as well as to positively modulate the regulation of serum cortisol and the activity of different neurotransmitters (Moyer et al., 2011; Hmwe et al., 2015; Zeng et al., 2016). Moreover, recent research highlight that manipulations may also influence the cytokine system and other pro-inflammatory molecules as well as cortical plasticity (Witzel et al., 2011; Yeh et al., 2017; Chang et al., 2020), which are known to be part of the pathomechanisms of neurodegenerative diseases (Bagyinszky et al., 2017; Meder et al., 2021).

Acupressure is non-invasive and can be performed by non-professionals at any time without the limitation of field or equipment. Once mastered, it can be practiced for life. Thus, acupressure may serve as a cost-effective, non-pharmacologic, complementary treatment for older adults with MCI. During this study, many participants reported significant improvements in overall health status through acupressure, such as sleep quality and emotion. Therefore, older adults are more likely to accept it and follow it for a long time.

Based on this study, we observed clear benefits for simultaneous acupressure and cognitive training over and above each modality alone, on the cognitive functions of older adults with MCI. Cognitive training is a process of active learning, which can fully tap the brain potential of the MCI patients and reconstruct the structure and function of the central nervous system (Sherman et al., 2017; Miotto et al., 2018; Hampstead et al., 2019). Beyond that, through the physical stimulation of acupressure at special acupoints, the cerebral blood flow of the corresponding cortex can be increased, the excitability of the nervous system can be regulated, the metabolism of the body can be increased, the immune function can be improved, and the brain atrophy can be prevented or slowed down (Yuan et al., 2019). Thus, the combination of cognitive training and acupressure combines their respective advantages and plays a joint role in promoting the benign changes in brain structure and function. However, from the evidence available, we have not come to a conclusion yet whether the combined effect is additive or synergistic. We did not collect brain imaging scans as part of this RCT and cannot address aspects of brain structure, function, and connectivity that underlie improvements in cognitive performance in response to simultaneous vs. individual intervening modalities. Determining how these combined acupressure and cognitive training interact should be a key element of future work.

The results of the present study showed that the improvements in cognition of participants in experimental groups emerged markedly in the first 3 months. At present, there is no unified length or duration of cognitive training or acupressure recommended for the treatment of MCI. Systematic reviews of cognitive training for MCI showed that the dose and duration of the cognitive training intervention was variable, with the total length of training ranging from 4 to 80 h and the duration of training from 2 to 24 weeks (Gates et al., 2019; Zhang et al., 2019a). In terms of acupressure, the evidence is very limited by now since there are few studies on the treatment of MCI by acupressure. Hence, more normative recommendation for appropriate length and duration of the interventions, as well as the frequency, needs to be developed in the future.

In the present study, cognitive training and acupressure can be feasibly performed by trained older adults with MCI. Self-administered training programs are essential. Not only do some older adults have limited time or ability to attend training programs away from their homes, but training programs require involvement by trainers who may not always be available or affordable (Bailey et al., 2010). Thus, widespread dissemination of a training technique will require self-administration by older adults. Results of previous studies support the efficacy of self-administered cognitive training in reliably improving cognitive and functional abilities, as well as self-efficacy (West et al., 2008), in normal older adults (Rizkalla, 2018) or individuals with MCI (Han et al., 2017). A systematic review of self-acupressure suggests that self-administered acupressure shows promise to alleviate the symptoms of various health problems, including allergic disease, cancer, respiratory disease, dysmenorrhea, perceived stress, insomnia, and sleep disturbances (Song et al., 2015). Moreover, cognitive training and acupressure can be self-administered without expensive costs, irrespective of geographic location, clinic and/or therapist availability. However, whilst the findings support the potential for self-administered cognitive training and acupressure as treatment strategy, they also underscore the importance of compliance monitoring in the delivery of the training, which needs to be carefully considered and planned before studies begin. Overall, under effective monitoring, self-administered training might be an innovative and effective strategy for older adults that remain independent and actively engaged in their brain health.

## Limitations

The present study had several limitations. First, the follow-up effect was not evaluated to detect the long-term sustainable efficacy of acupressure and cognitive training. Further researches with follow-up effect evaluation are therefore recommended. Second, although subjects who had suffered from diseases like depression or severe sleep disorder which could potentially compromise their cognition were excluded as eligible participants, there still might be some confounding factors that influenced the intervention effects during the trial. Third, the assessment of cognitive functioning was limited in this study, therefore more complete measurement tools and the consideration of including measurement of other variables such as quality of life were recommended for future studies. Fourth, the participants were not blind to their interventions, which may have led to a Hawthorne effect-like influence on the experimental groups. In addition, social interactions during the training might have positive cognitive effects on the participants. Thus, the lack of an active control which could replicate the social interactions was also a limitation of this study and need to be addressed in future studies.

## Conclusion

This study indicated that acupressure and cognitive training both could improve the cognitive functions of older adults with MCI, and when used together, the effects were enhanced. To the best of our knowledge, this is the first randomized controlled trial to evaluate the combined effect of acupressure and cognitive training on cognitive functions of older adults with MCI. Acupressure is safe, cost-effective and can be implemented by non-professionals. Thus, it may serve as a complementary intervention to improve cognitive functions and prevent dementia in community-dwelling older people, especially for those with MCI. Acupressure can be combined with other interventions such as cognitive training to achieve better improvements on cognitive functions.

## Data Availability Statement

The original contributions presented in the study are included in the article/supplementary material, further inquiries can be directed to the corresponding author/s.

## Ethics Statement

The studies involving human participants were reviewed and approved by China Ethics Committee of Registering Clinical Trials and Central South University. The patients/participants provided their written informed consent to participate in this study.

## Author Contributions

JS: methodology, project administration, investigation, and writing—original draft preparation. HZ: conceptualization, supervision, and writing—reviewing and editing. LP: investigation and formal analysis. XW: investigation and formal analysis. ML: investigation. All authors contributed to the article and approved the submitted version.

## Conflict of Interest

The authors declare that the research was conducted in the absence of any commercial or financial relationships that could be construed as a potential conflict of interest.

## Publisher’s Note

All claims expressed in this article are solely those of the authors and do not necessarily represent those of their affiliated organizations, or those of the publisher, the editors and the reviewers. Any product that may be evaluated in this article, or claim that may be made by its manufacturer, is not guaranteed or endorsed by the publisher.
